# 

**Published:** 1907-04-06

**Authors:** 


### BOOKS RECEIVED.

T. Fisher Unwin.

"Animal Micrology." By M. F. Guyer, Ph.D.

J. and A. Churchill.

"Text-Book of Anatomy for Nurses." By Elizabeth R. Bundy, M.D.

Kegan Paul, Trench, Trübner and Co., Ltd.

"Town Moods." By Oswald Davis.

Baillière, Tindall, and Cox.

"Antiseptic Methods." By Harold Upcott, F.R.C.S.

"The Röntgen Rays in Medical Work." By D. Walsh, M.D.

J. Wright and Co., Bristol.

"Medical Annual," 1907.

"Lessons on the Care of Infants." By Mrs. Watson.

Cassell and Co.

"Elements of Physics for Medical Students." By F. J. M. Page, B.Sc.

Hodder and Stoughton.

"Pathology" (Medical Epitome Series). By J. Stenhouse, M.A., and J. Ferguson, M.A.

P. S. King and Son.

"The Infant, the Parent, and the State." By H. Llewellyn Heath, D.P.H.

Sidney Appleton.

"Tics and their Treatment." By Hy. Meige and E. Feindel. Translated by S. A. K. Wilson, M.B.

**Notice to Advertisers and Subscribers.**

All Advertisements, Orders for Copies of The Hospital, and Business Communications should be addressed to **THE MANAGER (Not to the Editor),** The Hospital, 28 & 29 Southampton Street, Strand London, W.C.

The Cost of Subscription, post free, is as follows:—Per week, 3½d.; per quarter, 3s. 3d.; per half-year, 6s. 6d.; per annum, 13s. Foreign and Colonial:—Per annum, 19s. 6d., payable, in all cases, in advance.

